# WE DID MORE DAMAGE: HOW COVID-19 COLLAPSED THE CONVOYS OF CARE FOR RESIDENTS LIVING WITH DEMENTIA

**DOI:** 10.1093/geroni/igac059.1379

**Published:** 2022-12-20

**Authors:** Sara Hackett, Lindsay Peterson, Carlyn Vogel, Debra Dobbs

**Affiliations:** University of South Florida, Tampa, Florida, United States; University of South Florida, Tampa, Florida, United States; University of South Florida, Tampa, Florida, United States; University of South Florida, Tampa, Florida, United States

## Abstract

COVID-19 has posed a multitude of challenges for nursing homes (NHs) and assisted living communities (ALCs). However, little information is known about how the pandemic impacted residents living with dementia. Using the convoys of care framework, we conducted a qualitative descriptive study to gain insight regarding administrators’ perceptions of how care for residents living with dementia was altered. Forty-two participants, representing 16 NHs and 43 ALCs in Florida, participated in one 60-minute semi-structured interview. Thematic analysis revealed that the convoys of care for those living with dementia were strained by the competing demands associated with maintaining residents’ social and medical care. Explicitly, participants emphasized how diminished family involvement, the increasing responsibilities of staff, and the climate of the industry, contributed to disrupted convoys of care. Consequently, this study may help inform policy and practice as it details which care strategies worked, which did not, and considerations for moving forward.

